# MX2: Identification and systematic mechanistic analysis of a novel immune-related biomarker for systemic lupus erythematosus

**DOI:** 10.3389/fimmu.2022.978851

**Published:** 2022-08-18

**Authors:** Xiang-Wen Meng, Zhi-Luo Cheng, Zhi-Yuan Lu, Ya-Nan Tan, Xiao-Yi Jia, Min Zhang

**Affiliations:** ^1^ School of Pharmacy, Anhui University of Chinese Medicine, Hefei, China; ^2^ Anhui Province Key Laboratory of Chinese Medicinal Formula, Hefei, China; ^3^ Anhui Province Key Laboratory of Research and Development of Chinese Medicine, Hefei, China; ^4^ Department of Rheumatology and Immunology, The First Affiliated Hospital of USTC, Division of Life Sciences and Medicine, University of Science and Technology of China, Hefei, China

**Keywords:** systemic lupus erythematosus, MX2, machine learning, biomarker, immune infiltration

## Abstract

**Background:**

Systemic lupus erythematosus (SLE) is an autoimmune disease that involves multiple organs. However, the current SLE-related biomarkers still lack sufficient sensitivity, specificity and predictive power for clinical application. Thus, it is significant to explore new immune-related biomarkers for SLE diagnosis and development.

**Methods:**

We obtained seven SLE gene expression profile microarrays (GSE121239/11907/81622/65391/100163/45291/49454) from the GEO database. First, differentially expressed genes (DEGs) were screened using GEO2R, and SLE biomarkers were screened by performing WGCNA, Random Forest, SVM-REF, correlation with SLEDAI and differential gene analysis. Receiver operating characteristic curves (ROCs) and AUC values were used to determine the clinical value. The expression level of the biomarker was verified by RT‒qPCR. Subsequently, functional enrichment analysis was utilized to identify biomarker-associated pathways. ssGSEA, CIBERSORT, xCell and ImmuCellAI algorithms were applied to calculate the sample immune cell infiltration abundance. Single-cell data were analyzed for gene expression specificity in immune cells. Finally, the transcriptional regulatory network of the biomarker was constructed, and the corresponding therapeutic drugs were predicted.

**Results:**

Multiple algorithms were screened together for a unique marker gene, MX2, and expression analysis of multiple datasets revealed that MX2 was highly expressed in SLE compared to the normal group (all P < 0.05), with the same trend validated by RT‒qPCR (P = 0.026). Functional enrichment analysis identified the main pathway of MX2 promotion in SLE as the NOD-like receptor signaling pathway (NES=2.492, P < 0.001, etc.). Immuno-infiltration analysis showed that MX2 was closely associated with neutrophils, and single-cell and transcriptomic data revealed that MX2 was specifically expressed in neutrophils. The NOD-like receptor signaling pathway was also remarkably correlated with neutrophils (r >0.3, P < 0.001, etc.). Most of the MX2-related interacting proteins were associated with SLE, and potential transcription factors of MX2 and its related genes were also significantly associated with the immune response.

**Conclusion:**

Our study found that MX2 can serve as an immune-related biomarker for predicting the diagnosis and disease activity of SLE. It activates the NOD-like receptor signaling pathway and promotes neutrophil infiltration to aggravate SLE.

## Introduction

Systemic lupus erythematosus (SLE), a systemic autoimmune disease characterized by abnormal activity of the immune system, is a serious threat to human health (1, 2). Recently, accumulating evidence has demonstrated that some biomarkers have important reference values for the early diagnosis and treatment of SLE. For instance, hsa_circ_0000479 and TCONS_00483150 are novel diagnostic markers for SLE (3, 4). PGLYRP2 and DTX1 can predict disease activity in SLE patients (5, 6). DKK-1 can be used as a biomarker for active lupus erythematosus nephritis (7). However, the current SLE-related biomarkers still lack sufficient sensitivity, specificity and predictive power for clinical application. Based on the pathological changes in the immune microenvironment, it is necessary to discover new immune-related biomarkers for SLE diagnosis and development.

MX2 (MX Dynamin Like GTPase 2), encodes a protein with nuclear and cytoplasmic forms and is a member of the dynamin family and large GTPase family. The nuclear form is localized in a granular pattern in the heterochromatin region below the nuclear envelope. In the present study, we found that MX2 is closely related to inflammation-related pathways. Interestingly, inflammation-related signals, such as Toll-like receptor, NF-κB and Nod-like receptor, also play an important role in the development of SLE. For instance, in SLE patients, loss of B-cell tolerance to autoantigens is controlled by Toll-like receptors in a cell-intrinsic manner (8). Toll-like receptor signaling inhibitory peptides ameliorate inflammation in animal models and human SLE (9). A recent study found that reduced TNFAIP3 and enhanced UBE2L3 synergistically activate NF-κB, thereby synergistically increasing lupus risk (10). Peli1 negatively regulates noncanonical NF-κB signaling to inhibit SLE (11). Curcumin attenuates murine lupus by inhibiting NLRP3 inflammatory vesicles (12). Dietary olive bitter glycosides and their acyl derivatives ameliorated the pristane-induced inflammatory response in peritoneal macrophages of SLE mice and murine lupus nephritis by inhibiting the NLRP3 inflammatory vesicle pathway (13, 14). Meanwhile, MX2 has been found to play an important role in immune-related diseases and immune cells, but the function of MX2 and its detailed regulatory mechanisms in SLE remain unclear (15–18).

**Figure 1:**
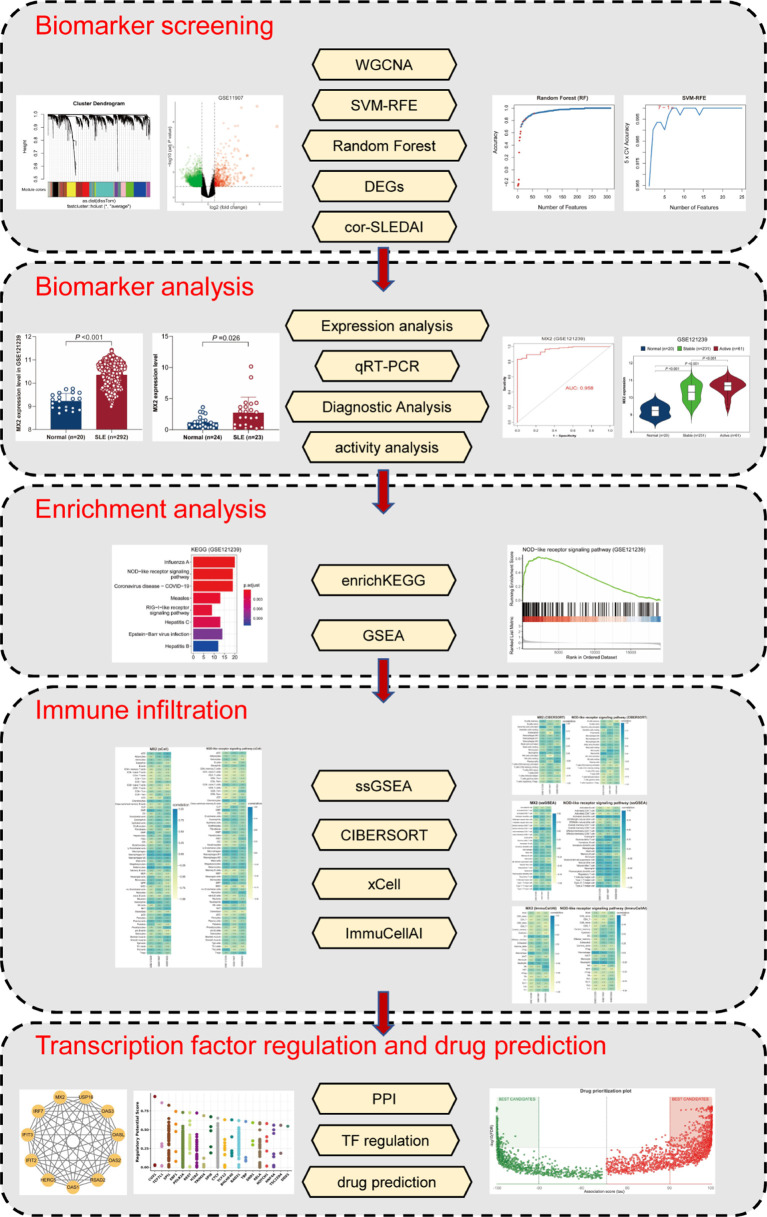
The flowchart of the analysis process.

In the present study, a total of five methods, namely, weighted gene co-expression network analysis (WGCNA), support vector machine-recursive feature elimination (SVM-RFE), random forest (RF), correlation with SLEDAI and differential expressed genes (DEGs) analysis, were initially applied to screen MX2, a potential biomarker for SLE. The differential expression levels of MX2 in SLE *vs.* healthy individuals were validated by real-time quantitative PCR (RT‒qPCR). Then, the relationship between MX2 and clinical parameters in SLE patients was analyzed. Thereafter, the mechanism by which MX2 promotes SLE was investigated by enrichKEGG and GSEA. Four immune infiltration analysis algorithms, including ssGSEA, CIBERSORT, xCell and ImmuCellAI, were performed to explore the relationship between MX2 and immune cells. Finally, the transcriptional regulatory network of MX2 was constructed, and the corresponding therapeutic drugs were predicted. The flowchart of this study was shown in **Figure 1**.

## Material and methods

### Data collection and processing

SLE-related public data were first downloaded from the Gene Expression Omnibus (GEO) database (19, 20), and seven datasets were collected: three datasets from PBMC samples [GSE121239 (GPL13158, Normal: 20, SLE: 292) (21, 22), GSE11907 (GPL96, Normal: 12, SLE: 110) (23), GSE81622 (GPL10558, Normal: 25, SLE: 30) (24)]; four datasets from whole blood samples [GSE65391 (GPL10558, Normal: 72, SLE: 924) (25), GSE100163 (GPL6884, Normal: 14, SLE: 55) (26–28), GSE45291 (GPL13158, Normal: 20, SLE: 292) (29), GSE49454 (GPL1261, Normal: 20, SLE: 157) (30)]. GEO2R was used for differential expression analysis of the GSE121239, GSE11907 and GSE81622 datasets. |log2 FC| > 0.5 and adj.P.Val <0.05 were screened as differentially expressed genes (DEGs). Information on the datasets was displayed in **Supplementary File S1**, and the UMAP plots of the datasets were shown in **Supplementary Figure S1**.

### Biomarker screening

Five methods were used for the screening of biomarkers of SLE: WGCNA (31), SVM-RFE and RF (32, 33) based on DEGs results from the GSE121239 dataset, SLEDAI correlation analysis, and the difference analysis of two SLE datasets (GSE11907, GSE81622). Gene module classification and identification of DEGs was performed using the R package “WGCNA” (34, 35). First, the samples were clustered to assess whether there were any significant outliers. Second, the automatic network construction function was used to construct the co-expression network. A soft threshold β is calculated. Third, hierarchical clustering and dynamic tree cutting functions were used to detect modules. Finally, gene importance (GS) and module membership (MM) were calculated. Linking modules to clinical traits. The corresponding module extracted the corresponding module gene information for further analysis. The SVM classifier was constructed using the R package “e1071” and cross-validated to eliminate regression features. The “randomForest” package was used for the RF classification process. For correlation analysis, the Spearman correlation coefficient was applied, and GEO2R was used for DEGs analysis of the SLE dataset (GSE11907, GSE81622). Then, overlapping genes were selected from the above five methods for further analysis in this study. The specific screening conditions for the analytic methods were shown in **Supplementary File S1**.

### PBMC isolation

A total of 47 samples, including 23 SLE samples and 24 healthy controls, were collected from the First Affiliated Hospital of USTC (Anhui Provincial Hospital). This study was approved by the ethics committee of the First Affiliated Hospital of USTC, and informed consent forms were signed by the patients. Peripheral blood mononuclear cells (PBMCs) were isolated from EDTA anticoagulated by FicollPaque density gradient centrifugation. Then, the cells were cultured in RPMI-1640 medium. Cells were maintained in a humidified incubator at 37°C with 5% CO_2_.

### Real-time quantitative PCR

RNA was extracted using total RNA isolation reagent (Epizyme, Shanghai, China). A cDNA synthesis kit (Bioer, Hangzhou, China) was used for reverse transcription of total RNA. GAPDH (forward: 5’-GTCTCCTCTGACTTCAACAGCG-3’, reverse: 5’-ACCACCCTGTTGCTGTAGCCAA-3’), MX2 (forward: 5’-CACCGAGCTAGAGCTTCAGGA-3’, reverse: 5’-CCGGGAAGGTCAATGATGGT-3’), and GAPDH was subsequently processed as internal references. Relative expression was calculated by the 2^-ΔΔCt^ method. *P <*0.05 was considered statistically significant.

### Functional enrichment analysis

Gene ontology (GO) and Kyoto Encyclopedia of Genes and Genomes (KEGG) pathway enrichment analysis were performed using the R package “clusterProfiler” (36) to explore the function and pathways of the screened genes. Gene set enrichment analysis (GSEA) (https://www.gsea-msigdb.org/gsea/index.jsp) was used to observe changes in pathways and biological processes in gene expression datasets. Additionally, the single-sample GSEA (ssGSEA) algorithm was utilized for the calculation of core pathway activity scores for each sample based on gene expression levels.

### Immune cell infiltration analysis

Four algorithms, ssGSEA, CIBERSORT, xCell and ImmuCellAI, were applied to perform immune cell infiltration analysis. The ssGSEA was performed using the R package “GSVA” to calculate the relative infiltration levels of 28 immune cell types (37). The xCell algorithm was implemented using the R package “xCell” to calculate the fraction of 64 immune cells in the sample. The CIBERSORT algorithm was applied to calculate the proportion of 22 immune cells in all samples. The standardized gene matrix was also used to estimate the abundance of immune cells in all samples through the ImmuCellAI website (http://bioinfo.life.hust.edu.cn/ImmuCellAI/#!/) (38). The results of immune infiltration were visualized by the R package “ggplot2” and the bioinformatics online website (http://www.bioinformatics.com.cn/). Violin plots were drawn to elucidate the expression levels of individual immune infiltrating cells among different subgroups. Correlation heatmaps were drawn to illustrate Spearman correlations of biomarkers or pathways with immune infiltrating cells. The CellMark database (http://bio-bigdata.hrbmu.edu.cn/CellMarker/index.jsp) was applied to query neutrophil-related marker genes (39). Based on the HPA database (40, 41) (https://www.proteinatlas.org/), transcriptional data and single-cell data were used to analyze the expression of biomarkers in immune cells.

### Transcription factor regulation and potential therapeutic drug prediction

The STRING database (https://cn.string-db.org/) was utilized to analyze the protein interaction relationships of biomarkers (42). The Cistrome DB database (http://cistrome.org/db/#/) was used to find transcription factors of the genes corresponding to these associated proteins (43). The DREIMT database (http://www.dreimt.org/) was applied to predict potential therapeutic drugs for SLE (44).

### Statistical analysis

All data processing and analysis were performed by R software (version 4.1.0) and GraphPad Prism (version 8.0.2). Differences between two groups of continuous non-normally distributed variables were analyzed using the Mann‒Whitney U test (Wilcoxon rank sum test). The Kruskal‒Wallis test was used for comparison of variables among multiple groups. *P <*0.05 was considered to be statistically significant.

## Result

### SLE biomarker screening

**Figure 2:**
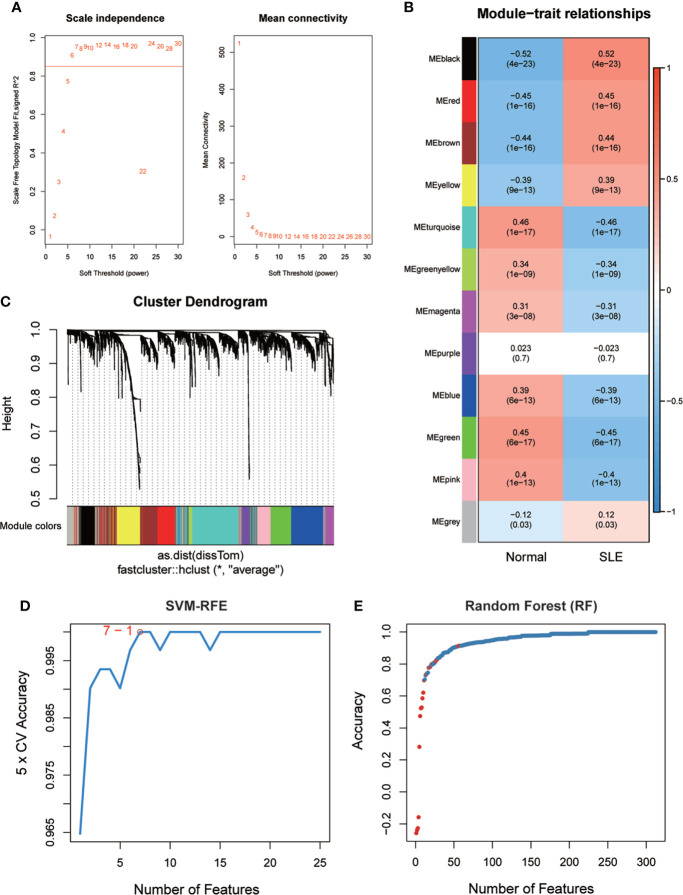
SLE biomarker screening. **(A)** Analysis of the scale-free fit index and the mean connectivity for various soft-thresholding powers. **(B)** Relationships of consensus modules with samples. It contains a set of highly linked genes. Each specified color represents a specific gene module. **(C)** Clustering dendrogram of differentially expressed genes related to SLE. **(D)**, **(E)** Based on support vector machine-recursive feature elimination (SVM -RFE) and random forest (RF) algorithm to screen biomarkers.

**Figure 3:**
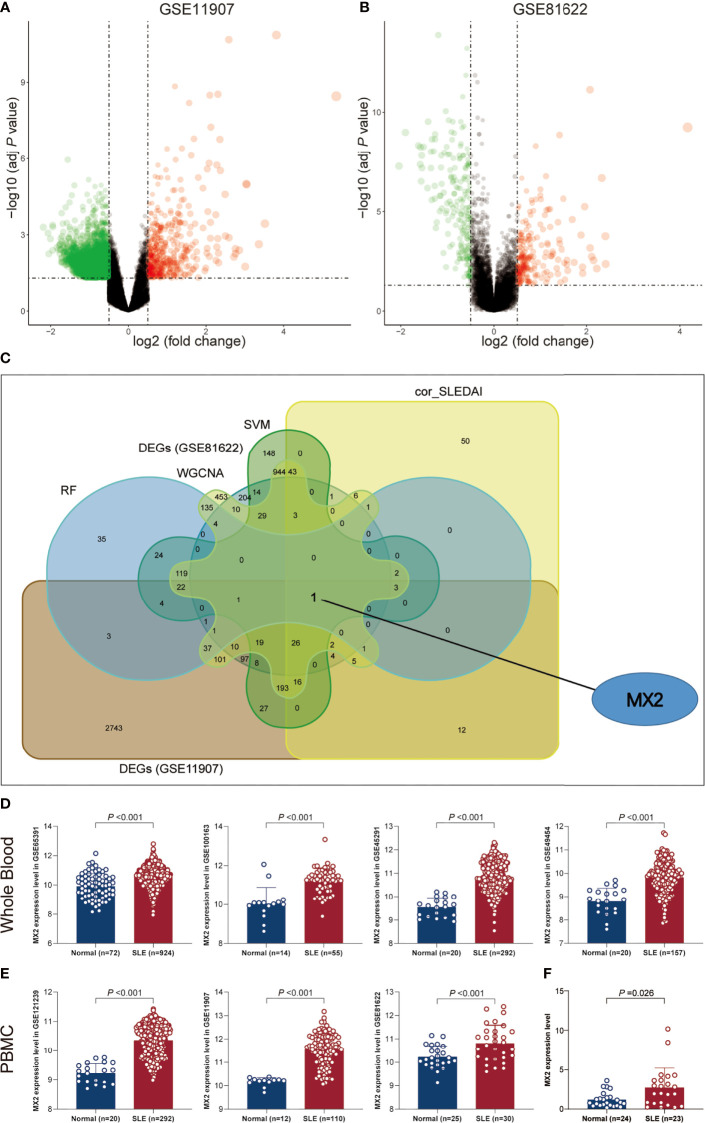
SLE biomarker screening. **(A)** Volcano plots of DEGs distribution in GSE11907. **(B)** Volcano plots of DEGs distribution in GSE81622. **(C)** Venn diagram showed the intersection of candidate biomarkers obtained by the five algorithms. **(D)** Expression levels of MX2 in SLE and normal donors in different whole blood datasets. **(E)** Expression levels of MX2 in SLE and normal donors in different PBMC datasets. **(F)** The MX2 expression was validated by qRT-PCR.

To identify potential biomarkers of SLE, five methods were applied. In the WGCNA algorithm, the sample clustering map was shown in **Supplementary Figure S2**. The soft threshold power was chosen as 30 when 0.85 was used as the correlation coefficient threshold (**Figure 2A**). The WGCNA network clustering analysis divided 2512 DEGs (GSE121239) into 12 modules, and then we performed correlation analysis on all modules and found eight modules closely associated with disease occurrence, with a total of 2181 genes (**Figure 2B**). The clustering dendrogram of DEGs associated with SLE was shown in **Figure 2C**. In total, 1646 genes were identified as key candidate biomarkers for SLE by the SVM-RFE algorithm (**Figure 2D**). In addition, 394 genes were screened as potential biomarkers using the RF algorithm (**Figure 2E**). Based on the correlation with SLEDAI, 176 potential key biomarkers were identified from the DEGs. In addition, 3337 and 435 DEGs were obtained from the GSE11907 and GSE81622 datasets, respectively (**Figures 3A, B**). We finally obtained the unique key biomarker MX2 by the above algorithms (**Figure 3C**).

### MX2 was highly expressed in SLE and served as a biomarker for diagnosis and disease activity

**Figure 4:**
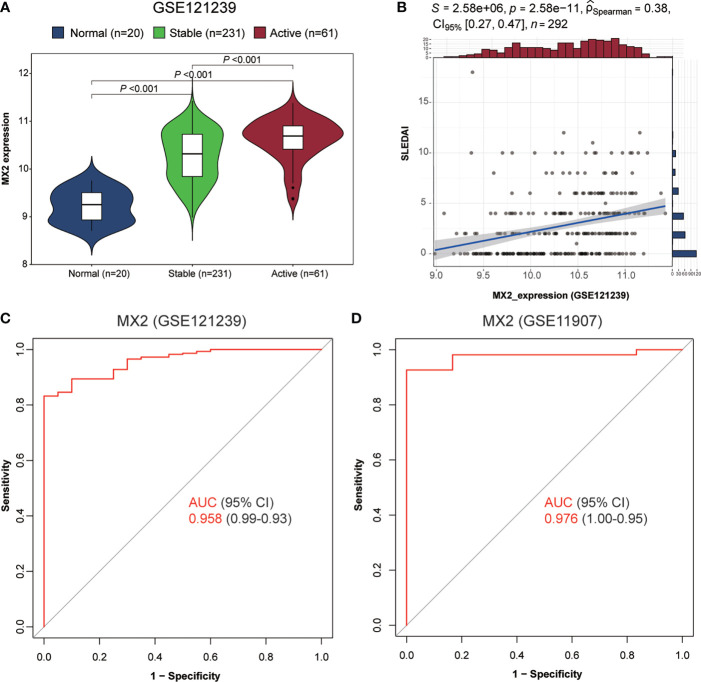
MX2 was highly expressed in SLE and served as a biomarker for diagnosis and disease activity. **(A)** Comparison of MX2 expression in normal and different stages of SLE. **(B)** Correlation of MX2 with SLEDAI. **(C)** The ROC curve for diagnostic efficacy validation of MX2 in GSE121239. **(D)** The ROC curve for diagnostic efficacy validation of MX2 in GSE11907.

As shown in **Figures 3D, E**, we found high MX2 expression in SLE based on multiple PBMC and whole blood datasets. Subsequently, the same result was validated by RT‒qPCR (**Figure 3F**). Further exploration of the clinical value revealed that the expression of MX2 was positively correlated with SLE severity and SLEDAI (*r* = 0.38, *P* < 0.05, **Figures 4A, B**). ROC curves showed the probability of MX2 as a valuable biomarker with AUCs of 0.958 and 0.976, respectively (**Figures 4C, D** **)**, with high accuracy.

### MX2 activated the NOD-like receptor signaling pathway in SLE

**Figure 5:**
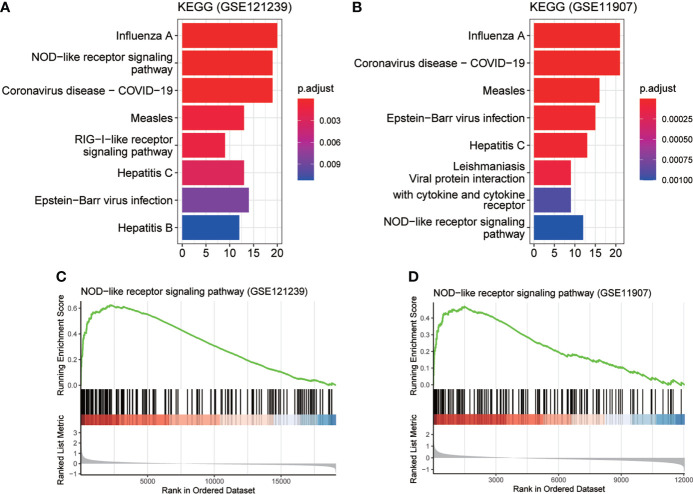
MX2 activated NOD-like receptor signaling pathway in SLE. **(A)**, **(B)** enrichKEGG pathway analysis results of MX2. **(C)**, **(D)** GSEA analysis results of MX2.

**Figure 6:**
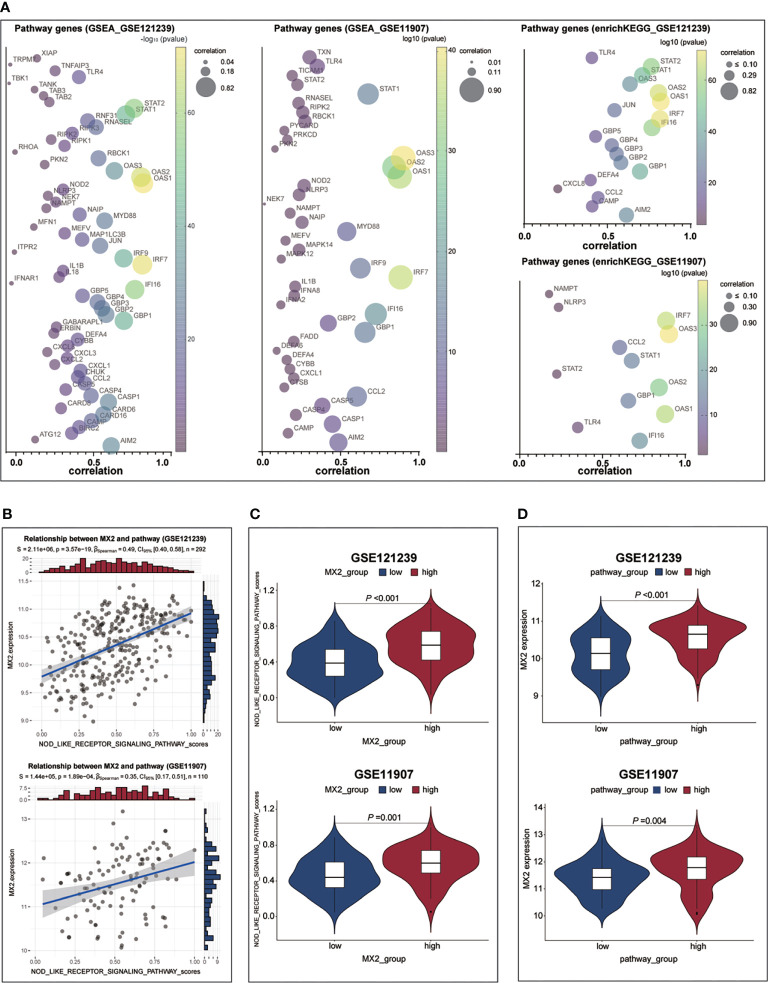
MX2 activated NOD-like receptor signaling pathway in SLE. **(A)** Correlation between MX2 and NOD-like receptor signaling pathway genes. **(B)** Correlation between MX2 and NOD-like receptor signaling pathway scores. **(C)** Comparison of the distribution of NOD-like receptor signaling pathway scores between high and low MX2 expression subgroups. **(D)** Comparison of MX2 expression between high and low subgroups of the NOD-like receptor signaling pathway scores.

To determine the detailed mechanisms of MX2 in SLE, we performed a multifaceted analysis and validation. enrichKEGG analysis showed that MX2 was mainly enriched in the NOD-like receptor signaling pathway, and the same results were deduced from the GSE11907 and GSE81622 datasets (**Figures 5A, B** and **Supplementary Figure S3C**). The GSEA of the above three datasets also identified the NOD-like receptor signaling pathway as an important association pathway of MX2 (**Figures 5C, D** and **Supplementary Figure S3D**). To further verify the relationship between MX2 and the NOD-like receptor signaling pathway, we performed correlation analysis between MX2 and genes of this pathway. The results showed that MX2 was correlated with most genes of the pathway (**Figure 6A**, **Supplementary Figures S4A, B**). Among them, OAS1, OAS2, OAS3, STAT1, and IRF9 showed a high correlation with MX2 in all three datasets (**Figure S4C**). Next, the ssGSEA algorithm was used to score the NOD-like receptor signaling pathway for the SLE samples. Based on the score, the correlation between MX2 and the NOD-like receptor signaling pathway was calculated (**Figure 6B**, **Supplementary Figure S4D**), and the results showed that MX2 was clearly correlated with the NOD-like receptor signaling pathway. There were significant differences in pathway scores between the high and low MX2 expression subgroups (**Figure 6C**, **Supplementary Figure S4E**) and similarly significant differences in MX2 expression between the high and low pathway score subgroups (**Figure 6D**, **Supplementary Figure S4F**).

### MX2 was mainly positively associated with neutrophil infiltration

**Figure 7:**
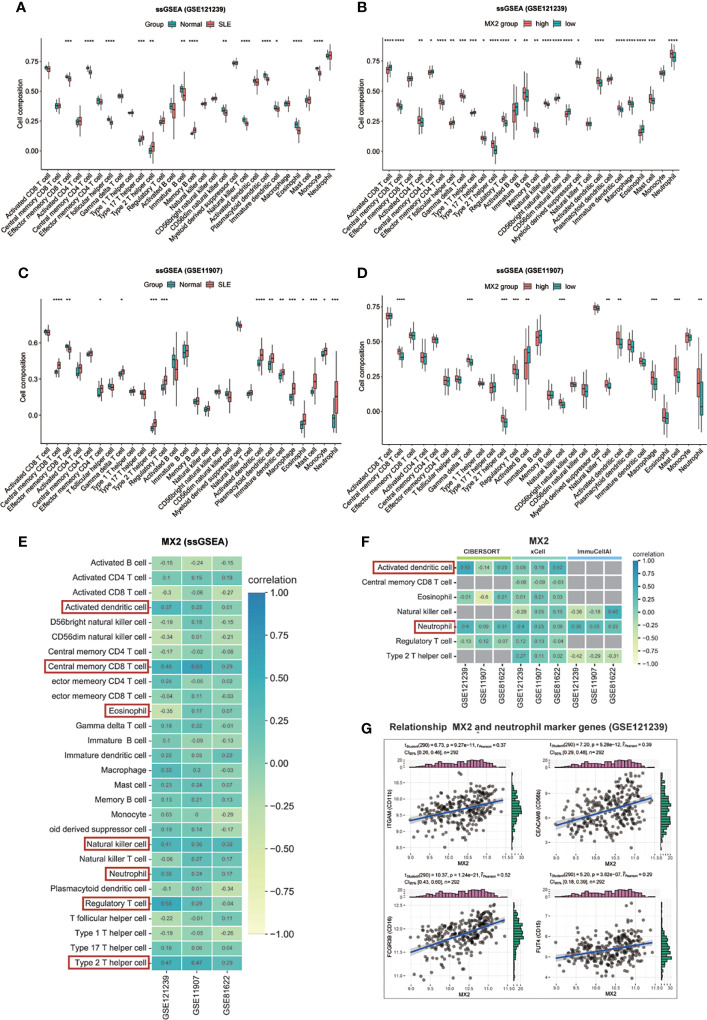
MX2 was mainly positively associated with neutrophil infiltration. **(A)**, **(C)** Comparison of the ssGSEA scores for immune cells estimated between all samples with Normal and SLE in the GSE121239. **(B)**, **(D)** Comparison of the ssGSEA scores for immune cells estimated between patients with high and low MX2 expression subgroups in the GSE121239. **(E)** Correlation between MX2 and infiltrating immune cells in three datasets (ssGSEA). **(F)** Correlation between MX2 and seven types of infiltrating immune cells in three datasets (CIBERSORT, xCell and ImmuCellAI). The gray area indicates that the algorithm does not contain this type of cell. **(G)** Correlation between MX2 and neutrophil marker genes. (*P <0.05, **P <0.01, ***P <0.001, ****P <0.0001).

**Figure 8:**
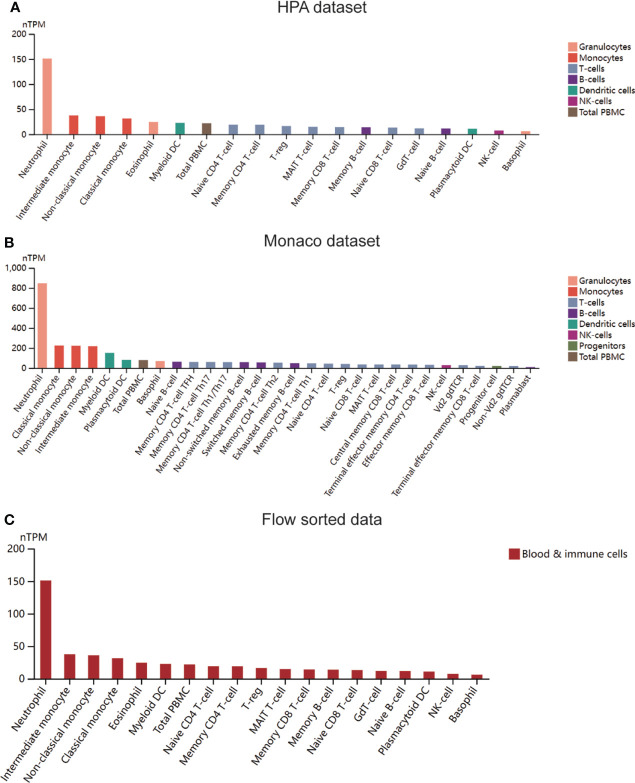
MX2 was mainly positively associated with neutrophil infiltration. **(A)** Expression of MX2 in immune cells in the HPA dataset. **(B)** Expression of MX2 in immune cells in the Monaco dataset. **(C)** Expression of MX2 in immune cells in the flow sorted data from HPA database.

Four immune infiltration analysis methods were employed to explore the relationship between MX2 and immune cells. Based on the results of ssGSEA, 28 differences in immune cells between the normal and SLE groups were shown in **Figures 7A, C**, and differences in immune cells between the high and low MX2 expression subgroups were shown in **Figures 7B, D**. Correlation analysis between MX2 and immune cells showed that MX2 was associated with activated dendritic cells, central memory CD8 T cells, eosinophils, natural killer T cells, neutrophils, regulatory T cells and type 2 T helper cells with a high correlation (**Figure 7E**). Further validation analysis of these seven immune cells by CIBERSORT, xCell and ImmuCellAI algorithms revealed that the relationship between MX2 and activated dendritic cells and neutrophils also remained consistent in these three algorithms, and the trend of neutrophils was much more stable than that of activated dendritic cells (**Figure 7F**). The results of immune cells infiltration analysis of all CIBERSORT, xCell and ImmuCellAI were presented in **Supplementary Figures S5**, **S6A–C**. Based on the CellMark database, the correlation of MX2 with neutrophil marker genes was analyzed (**Figure 7G**), and the results showed that MX2 correlated with all the neutrophil marker genes. In immune cell analysis using the HPA database, HPA and Monaco transcriptome data showed that MX2 was predominantly expressed in neutrophils (**Figures 8A, B**), and gene expression clustering data showed that MX2 was part of the cluster 50 (neutrophil-cellular senescence) gene cluster with a confidence level of 0.98129 (**Supplementary Figure S6D**). Then, in single-cell analysis, the flow cytometry data showed that MX2 in PBMCs was mainly expressed in neutrophils (**Figure 8C**), and the gene expression clustering data showed that MX2 was part of the cluster of cluster 19 (monocytes and neutrophils-innate immune response)-related genes with a confidence level of 1 (**Supplementary Figure S6E**).

### Activation of the NOD-like receptor signaling pathway promoted neutrophil infiltration

**Figure 9:**
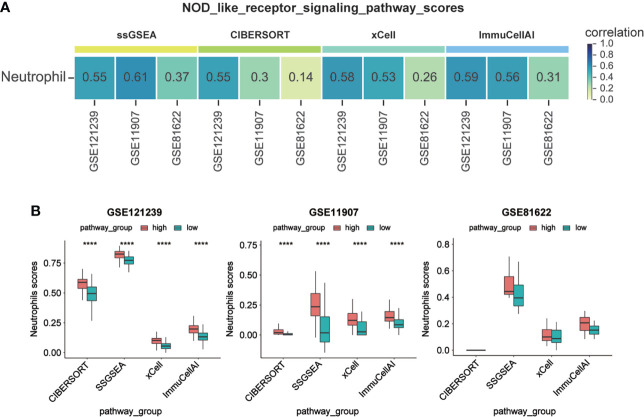
Activation of NOD-like receptor signaling pathway promoted neutrophil infiltration. **(A)** Correlation between NOD-like receptor signaling pathway scores and Neutrophil infiltration scores. **(B)** Comparison of Neutrophil infiltration scores between high and low subgroups of the NOD-like receptor signaling pathway scores. (****P <0.0001).

To explore the relationship between MX2, the NOD-like receptor signaling pathway and neutrophils, based on four immune infiltration analysis algorithms and correlation analysis, we found that the NOD-like receptor signaling pathway was significantly associated with neutrophils (**Figure 9A**). The difference in neutrophils between the high and low pathway groups was shown in **Figure 9B**. The results showed that neutrophil scores were significantly higher in the high NOD-like receptor signaling pathway scoring group in the GSE121239 and GSE11907 datasets. The same trend was observed in the GSE81622 dataset. The results of the correlation of the NOD-like receptor signaling pathway with all immune cells of the four algorithms were shown in **Supplementary Figures S7A–D**.

### MX2 regulatory network

**Figure 10:**
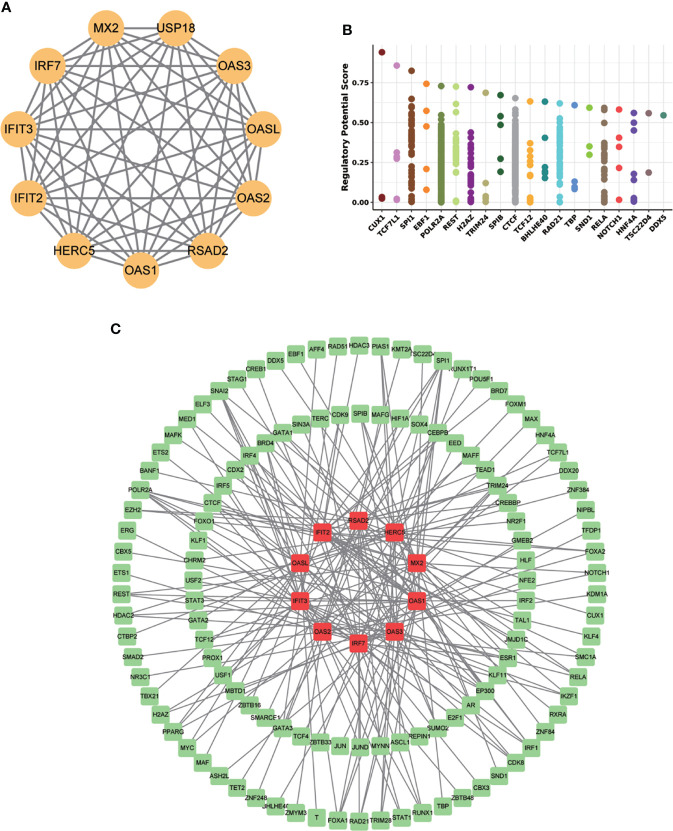
MX2 regulatory mechanism network. **(A)** The MX2 PPI network constructed by the STRING database. **(B)** The top 20 transcription factors regulating MX2 in the Cistrome DB database. **(C)** Protein-interacting gene-transcription factor (TF) network. Red represents genes and green represents TFs.

To determine the regulatory mechanism of MX2, we used the STRING database for PPI analysis to predict the proteins closely related to MX2, including HERC5, IFIT2, IFIT3, IRF7, MX2, OAS1, OAS2, OAS3, OASL and RSAD2 (**Figure 10A**). Besides, Cistrome DB database was applied to explore these transcription factors (TFs) of the corresponding genes of related proteins (**Figure 10B**, **Supplementary Figure S8**). The results showed that the TFs regulating MX2 mainly included CUX1, TCF7L1 and SPI1, and the “gene-transcription factor” regulatory network was established by Cytoscape, which contains 132 nodes and 200 edges (**Figure 10C**).

### Drug screening

**Figure 11:**
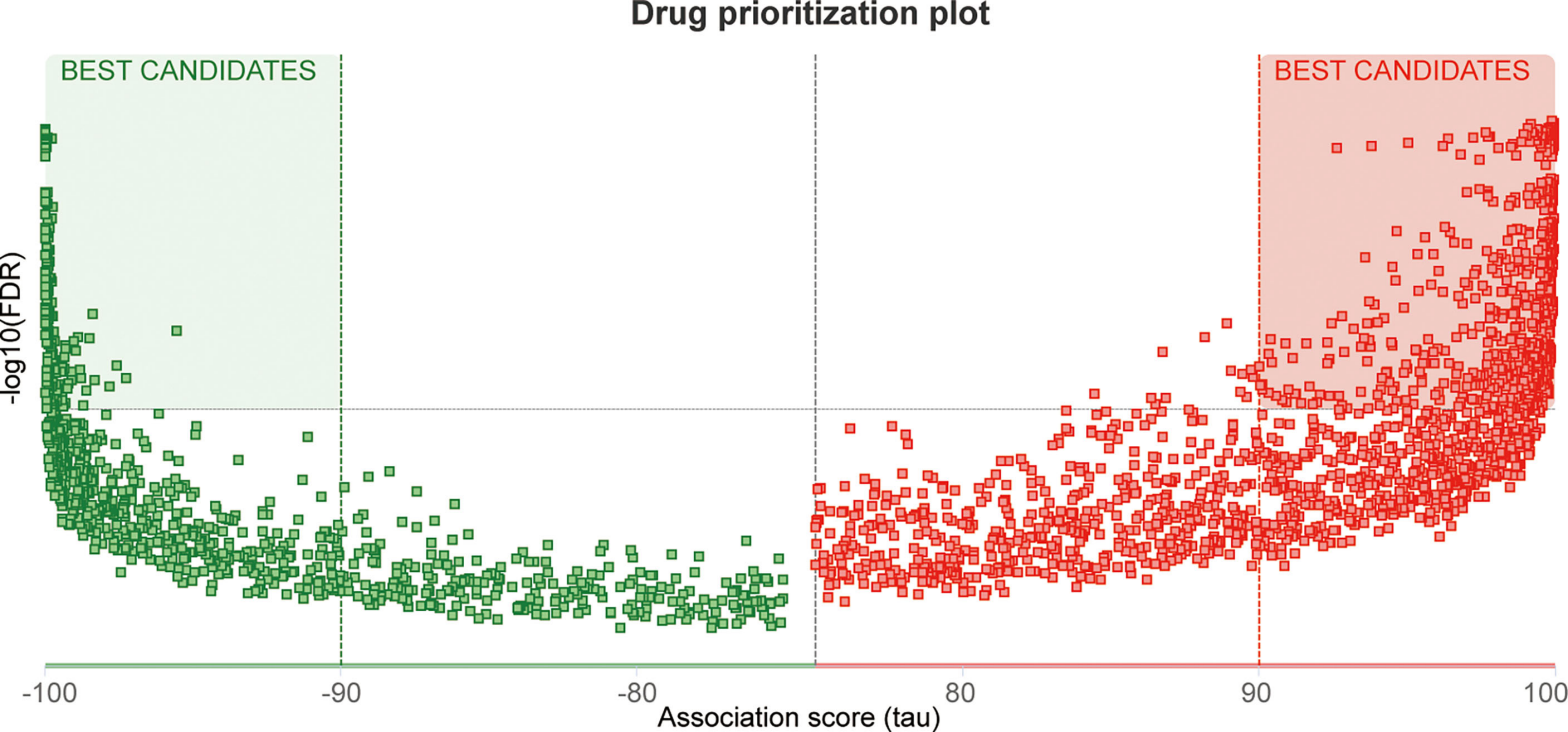
Drug screening. Heatmap of the distribution of potential therapeutic drugs.

A total of 24 genes, including MX2 and its highly related NOD-like receptor signaling pathway genes (*r >*0.5), were used as pathogenic targets for drug prediction, and the distribution of the screened drugs was shown in **Figure 11**, of which 652 drugs had |tau| scores >99, with detailed scores in **Supplementary File S6**.

## Discussion

MX2 plays an important role in immunity mainly against viruses, including HIV, hepatitis B and C viruses (45–47). Recently, MX2 has also been identified as an immune-related marker in rheumatoid arthritis (RA) (15). It is involved in immune regulation by several molecular interactions, including induction by IFN and consequent inhibition of HIV-1 (16). It can exert antiviral effects through IRF-3 and IRF-7 without involving IFN-γ signaling (48). In addition, MX2 may also modulate XAF1 to increase the sensitivity of melanoma cells to targeted therapies, which may be related to immune responses (49). Moreover, MX2 exerts immune effects by mediating multiple immune cells. Zhao et al. (17) found that MX2 is involved in the construction of models associated with neutrophils, macrophages, CD4^+^ T cells and CD8^+^ T cells in endometrial cancer. MX2 regulates SAMHD1 and thus prevents macrophages, resting CD4^+^ T cells and dendritic cells from being infected by HIV-1 (18).

Although the role of MX2 in various immune-related diseases, such as AIDS, hepatitis C, and RA, has been previously studied, the relationship between MX2 and SLE has not been thoroughly investigated. In this study, we screened for MX2, and further analysis revealed that it showed high expression in both whole blood and peripheral blood datasets in SLE compared to normal samples. Thereafter, ROC curves showed that it was an important potential diagnostic marker for SLE, and it also showed a significant correlation with the degree of disease activity. Taken together, our results suggested that MX2 was a biomarker for the diagnosis and activity level of SLE.

Besides, we found that the activation of the NOD-like receptor signaling pathway was significantly enriched in the high MX2 expression SLE group using enrichKEGG and GSEA methods. Meanwhile, correlation analysis showed that MX2 was positively correlated with the NOD-like receptor signaling pathway. Further analysis of the differences in pathway scores between the high and low MX2 expression subgroups showed a positive correlation between MX2 and the NOD-like receptor signaling pathway. The same results were obtained for the differences in MX2 expression between the high- and low-scoring pathway subgroups. Recent studies have also reported an essential role of the NOD-like receptor signaling pathway and its pathway-related genes in SLE. Tan et al. (50) found that NLRP3 in SLE bone marrow-derived mesenchymal stem cells could be targeted by Let-7f-5p to improve inflammation. In addition, Yu et al. (51) found that the expression of NOD2 in monocytes and plasmacytoid dendritic cells of SLE patients was significantly higher than that in healthy cells. Bacterial exposure increased NOD2 expression in monocytes, leading to the production of proinflammatory cytokines by PBMCs and exacerbating the condition of SLE. Moreover, one study demonstrated that the NLRP1 rs2670660 SNP and NLRP1 rs12150220-rs2670660A-G haplotype increased the risk of SLE (52). In summary, all these findings suggest that the process of SLE exacerbated by MX2 expression is closely related to the activation of the NOD-like receptor signaling pathway, and the activation of this pathway may occur mainly through OASs.

As we known, SLE is an autoimmune disease and is closely related to immune cells. Therefore, we analyzed the relationship between MX2 and immune cells using four immune infiltration analysis algorithms, and the results showed that it was significantly associated with neutrophils. It also had a remarkable correlation with neutrophil marker genes. Meanwhile, transcriptome and single-cell data analysis showed that MX2 was specifically expressed in neutrophils based on the HPA database. Likewise, in a previous study, MX2 was proven to be involved in the construction of a model associated with neutrophils in endometrial cancer (17). The detection of MX2 and other related genes in blood neutrophils is crucial in the diagnosis and evaluation of pregnant cows (53–55). Thus, this evidence suggests that MX2 promotes SLE by increasing neutrophil infiltration, which may be a key factor in MX2 aggravating SLE.

Subsequently, we found that MX2 promotes SLE in relation to the NOD-like receptor signaling pathway and neutrophils. Accordingly, we further explored the relationship among these three. Through immune infiltration and correlation analysis, we found that MX2, the NOD-like receptor signaling pathway and neutrophils all showed positive correlations among SLE patients. Recently, a similar study also found that the NOD-like receptor signaling pathway promoted neutrophilic asthma (NA) (56). In major depressive disorder (MDD), neutrophil activation was associated with activation of the NOD-like receptor signaling pathway (57). Cai et al. (58) found that NLRP6 regulated neutrophil homeostasis in bacterial pneumonia-derived sepsis. These results suggested that MX2 may promote neutrophil infiltration through activation of the NOD-like receptor signaling pathway, which further exacerbates SLE disease progression with increased infiltration of inflammatory cells and elevated MX2 expression.

Finally, we performed a predictive analysis of the transcriptional regulation mechanism and possible therapeutic drugs sensitive to MX2 in SLE. Most of the MX2-interacting proteins, including OAS1, OAS2, OAS3, OASL, IFIT2, IFIT3, IRF7, RSAD2, USP18 and HERC5, were closely related to SLE. OAS1, OAS2, OAS3 and IFIT3 could be used as biomarkers of SLE (59–62). Meanwhile, OAS2, OAS3 and OASL can be upregulated by MALAT1, which is involved in type I IFN-mediated SLE (63). IRF7 was a functional factor associated with SLE (64). RSAD2 and IFIT2 were potential LN therapeutic molecules (65). Besides, our results showed that the top-ranked MX2-associated transcription factors were also associated with immune regulation, including CUX1 and SPI1. Kuhnemuth et al. (66) found that CUX1 regulated the polarization of tumor-associated macrophages by regulating NF-κB signaling. SPI1 had a key role in determining the prognosis of GC patients and may be a potential immunotherapeutic target (67). In addition, 652 high-scoring drugs were obtained in the drug prediction analysis, which provides a certain guiding value for the clinical treatment of SLE.

There were some limitations in our study. First, detailed treatment information of the patients from the public datasets was unknown, which may somewhat affect the predictive value of MX2. Second, this study used multiple algorithms for biomarker screening and immune infiltration analysis; however, these algorithms may have some differences between each other due to their different algorithmic principles. Finally, our study was a secondary mining of existing public datasets, and although the analysis was validated by multiple datasets against each other, further evidence from clinical trials with large samples was still needed.

## Conclusion

In conclusion, we screened for a novel immune-related biomarker, MX2, in SLE and validated its high expression in patients. The mechanism of MX2 promotion in SLE was elucidated to originate from the activation of the NOD-like receptor signaling pathway and infiltration of neutrophils. In addition, the present study provided potential therapeutic drugs for the clinical treatment of SLE based on MX2-related mediated genes.

## Data availability statement

Publicly available datasets were analyzed in this study. This data can be found here: GEO database (http://www.ncbi.nlm.nih.gov/geo/). The accession number(s) can be found in the article. Resulting data are available through **Supplementary Material**, and other reasonable requests can be obtained from the corresponding authors.

## Ethics statement

The studies involving human participants were conducted according to the guidelines of the Declaration of Helsinki, and reviewed and approved by the Medical Research Ethics Committee of The First Affiliated Hospital of USTC (2022-RE-148). The patients/participants provided their written informed consent to participate in this study.

## Author contributions

MZ and X-YJ designed the study. Project funding was provided by MZ. X-WM performed main microarray analysis and experimental validation. Z-LC and Z-YL participated in the screening and partial analysis of the public datasets. Y-NT confirmed the authenticity of all the raw data. MZ, X-YJ and X-WM edited the manuscript. All authors read and approved this final manuscript.

## Funding

This work was partly supported by the Natural Science Foundation of Anhui Province (No. 1808085MH298), the China Postdoctoral Science Foundation Grant (No. 2020M682051), and Anhui Province Postdoctoral Research Funding Program Project (No. 2021A480).

## Acknowledgments

The authors are grateful for the enthusiastic support of Dr. Wei Wang in the Department of Medical Oncology in the First Affiliated Hospital of USTC.

## Conflict of interest

The authors declare that the research was conducted in the absence of any commercial or financial relationships that could be construed as a potential conflict of interest.

## Publisher’s note

All claims expressed in this article are solely those of the authors and do not necessarily represent those of their affiliated organizations, or those of the publisher, the editors and the reviewers. Any product that may be evaluated in this article, or claim that may be made by its manufacturer, is not guaranteed or endorsed by the publisher.

## Supplementary material

Supplementary file 1 The information of datasets and screening for MX2.

Supplementary file 2 MX2 expression and clinical significance analysis data.

Supplementary file 3 MX2 enrichment analysis and pathway correlation analysis data.

Supplementary file 4 Correlation analysis data of MX2 and immune cells.

Supplementary file 5 Correlation analysis data between NOD-like receptor signaling pathway and immune cells.

Supplementary file 6 Drug predictive analytics data.

Supplementary Figure 7:1 The UMAP plot in the GEO datasets.

Supplementary Figure 7:2 The sample clustering map of GSE121239.

Supplementary Figure 7:3
**(A)** enrichGO analysis results of MX2. **(B)** gseGO analysis results of MX2. **(C)** enrichKEGG pathway analysis results of MX2. **(D)** GSEA analysis results of MX2.

Supplementary Figure 7:4
**(A)**, **(B)** Correlation between MX2 and NOD-like receptor signaling pathway genes. **(C)** Intersection of pathway genes with high correlation with MX2. **(D)** Correlation between MX2 and NOD-like receptor signaling pathway scores. **(E)** Comparison of the distribution of NOD-like receptor signaling pathway scores between high and low MX2 expression subgroups. **(F)** Comparison of MX2 expression between high and low subgroups of the NOD-like receptor signaling pathway scores.

Supplementary Figure 7:5 Calculation of the difference of immune cells between different groups based on xCell **(A)**, CIBERSORT **(B)**, ImmuCellAI **(C)** and ssGSEA **(D)** algorithms (normal vs. SLE, MX2_high vs. MX2_low).

Supplementary Figure 7:6 Calculation of the correlation between MX2 and immune cells based on CIBERSORT **(A)**, xCell (B) and ImmuCellAI **(C)** algorithms. **(D)**, **(E)** Gene expression cluster map based on HPA database.

Supplementary Figure 7:7 Calculation of the correlation between NOD-like receptor signaling pathway scores and immune cells based on ssGSEA **(A)**, xCell **(B)**, CIBERSORT **(C)** and ImmuCellAI **(D)** algorithms.

Supplementary Figure 7:8 Transcription factor prediction.
