# Supplementary figures and images for: MX2: Identification and systematic mechanistic analysis of a novel immune-related biomarker for systemic lupus erythematosus
Source: Front Immunol. 2022 Aug 18;13:978851. doi: 10.3389/fimmu.2022.978851 (PMC9433551; doi:10.3389/fimmu.2022.978851)

**S2**

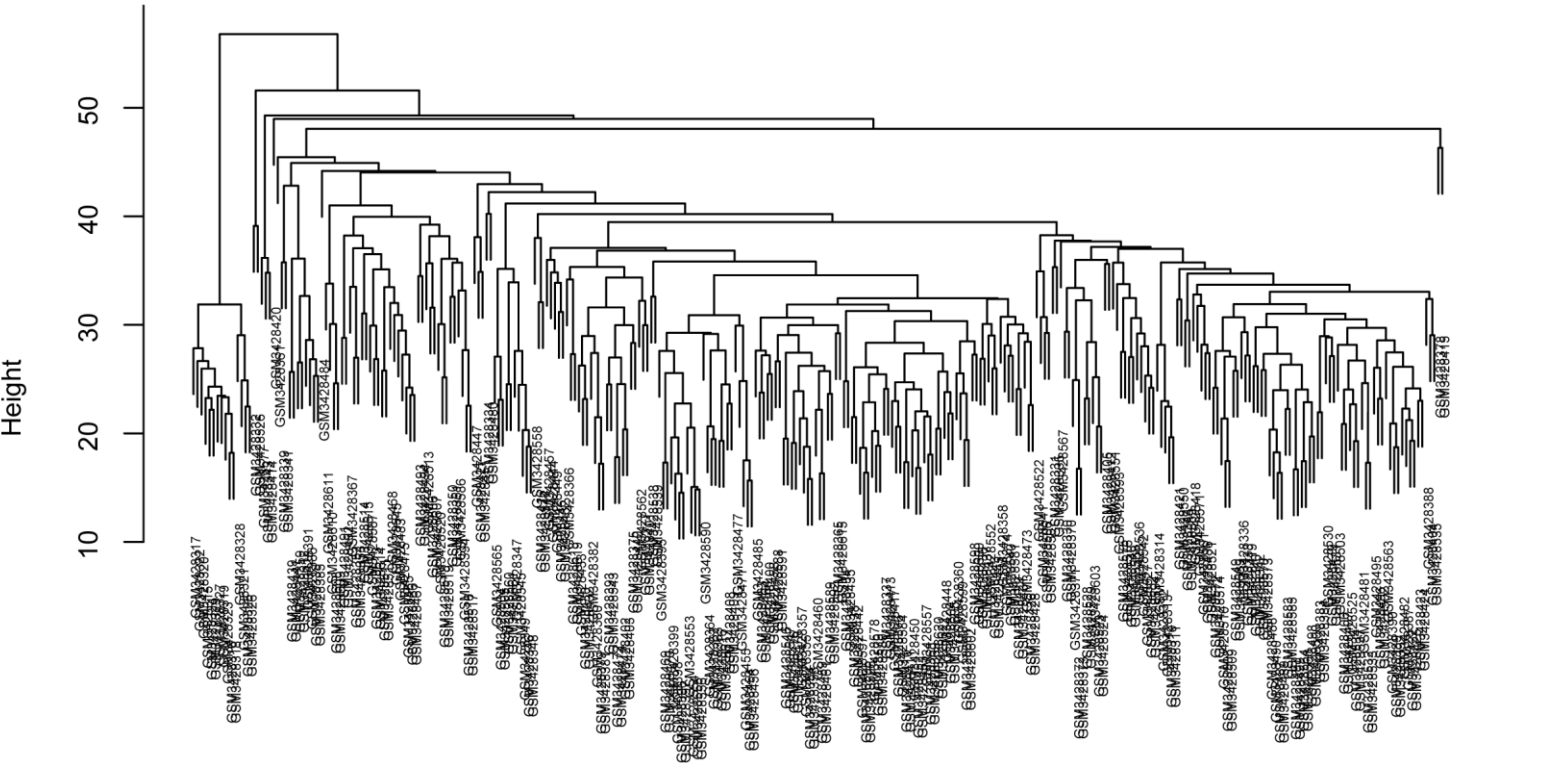

A

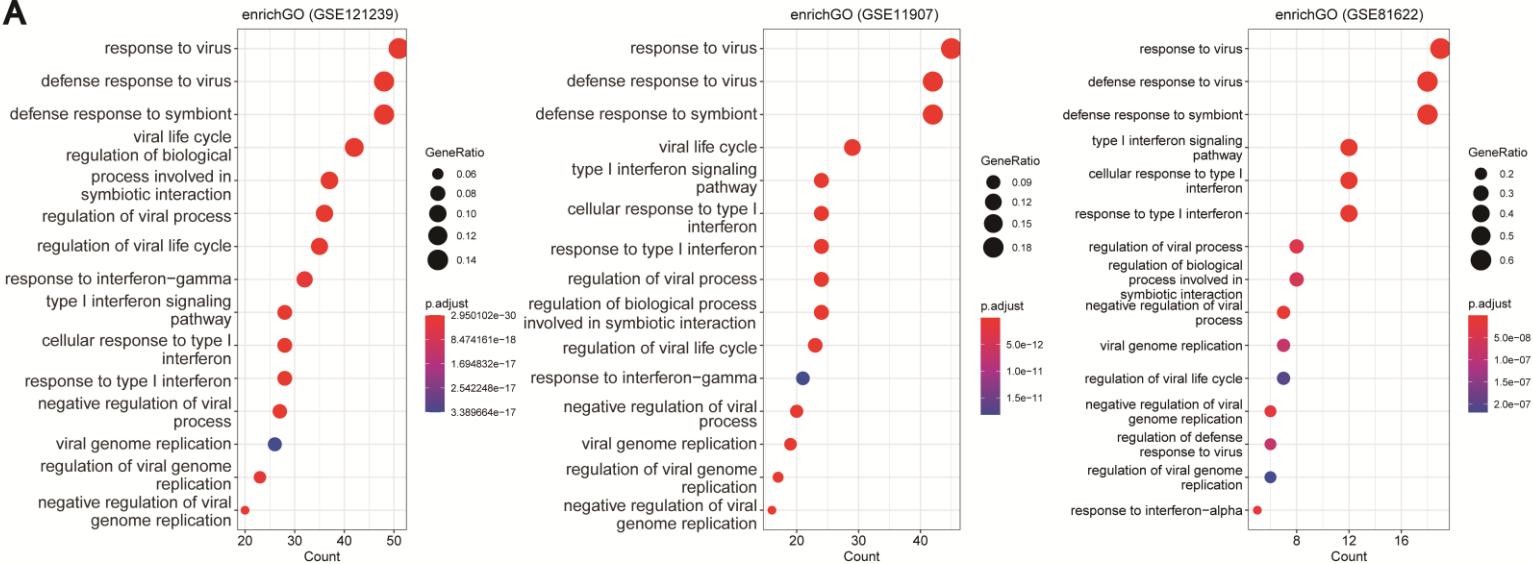

B

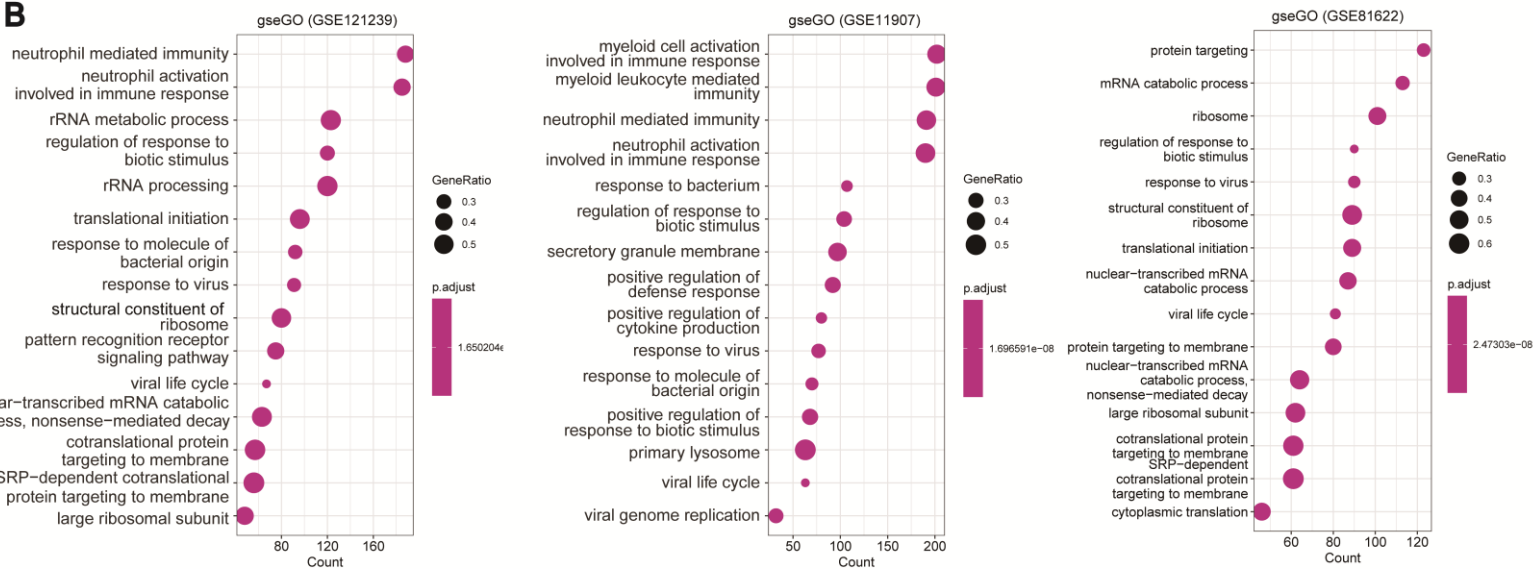

C

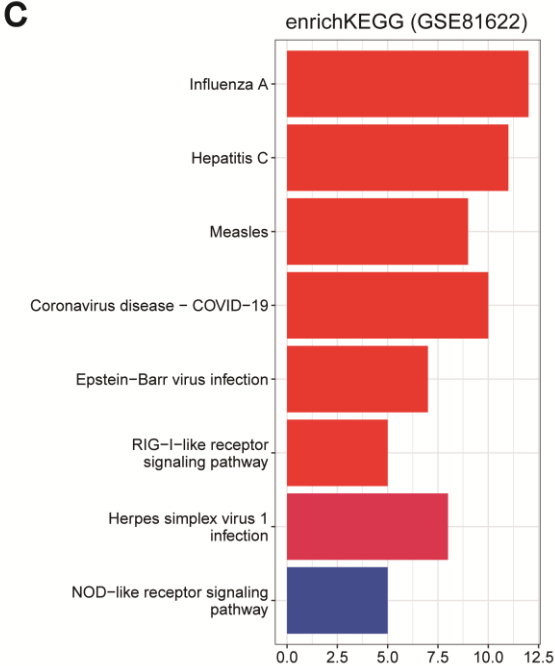

D

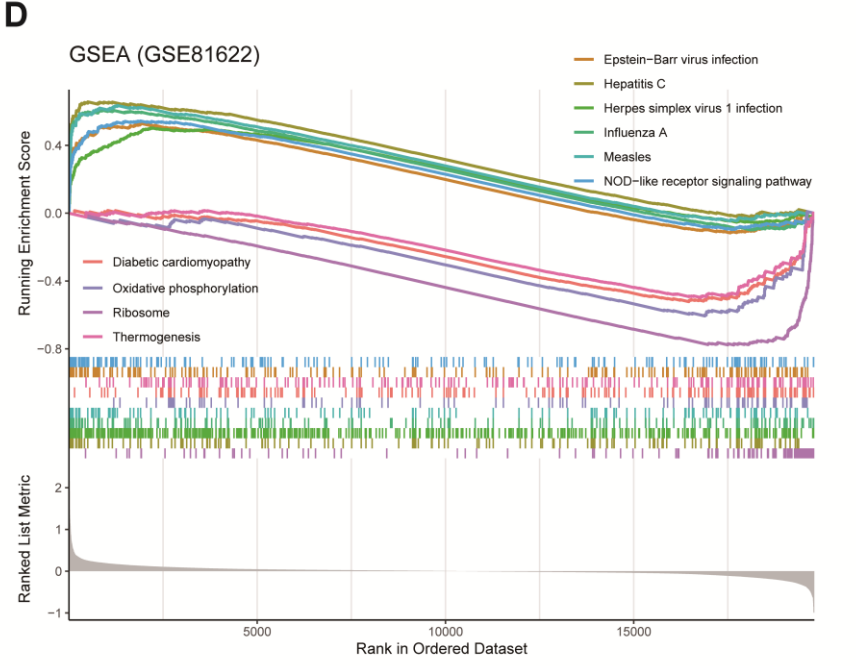

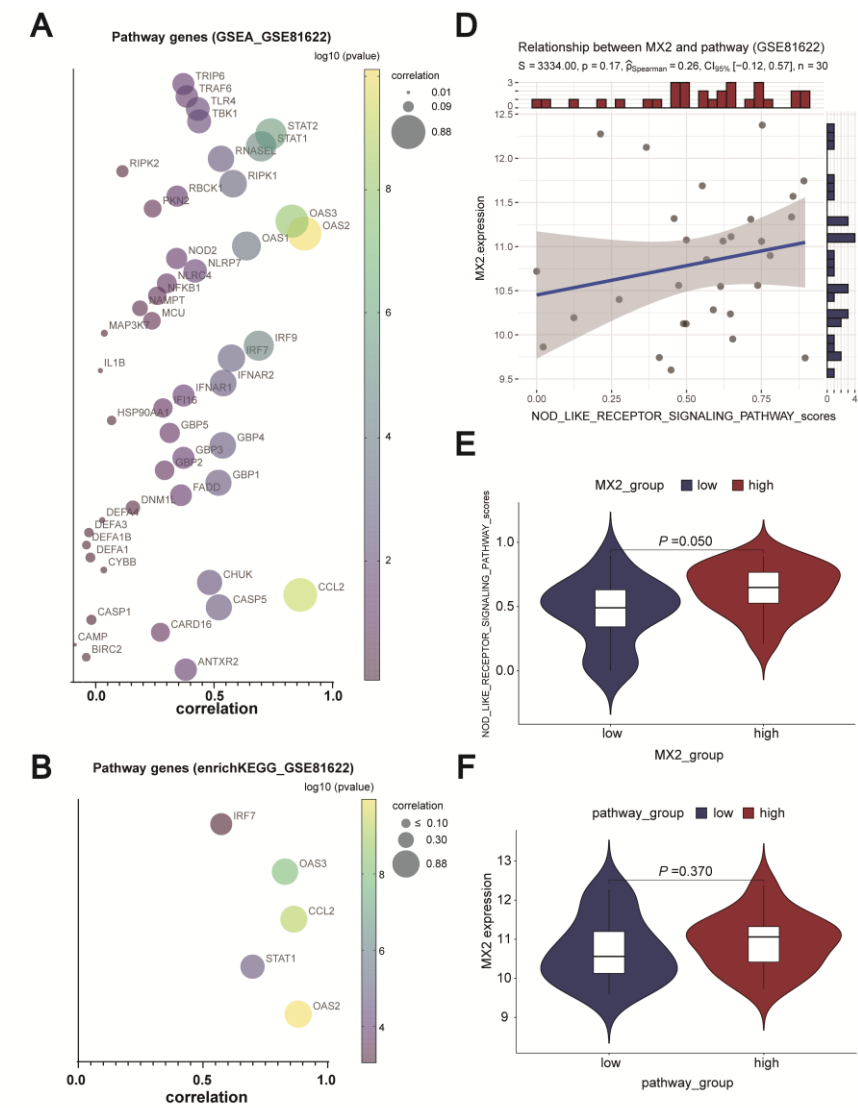

A

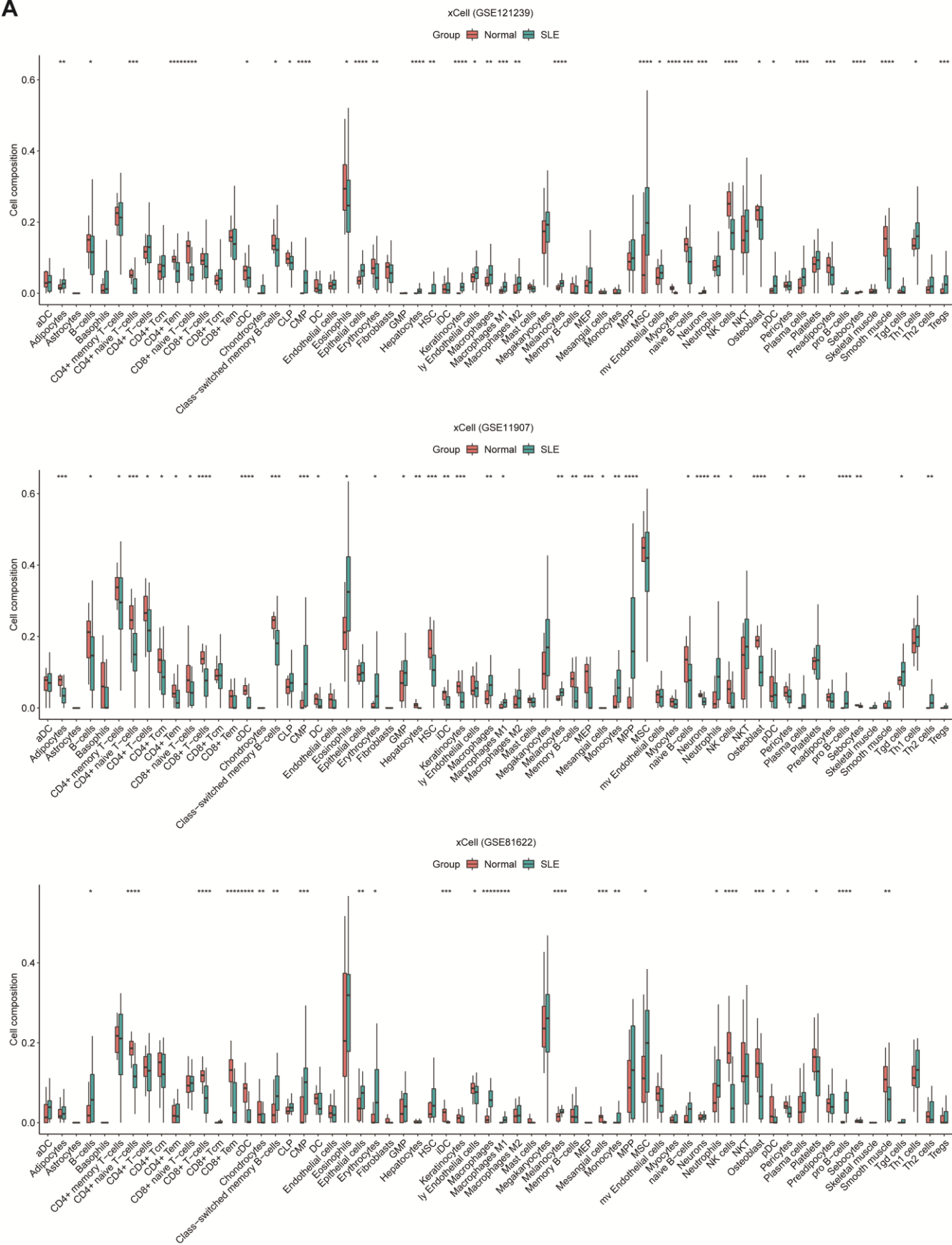





A

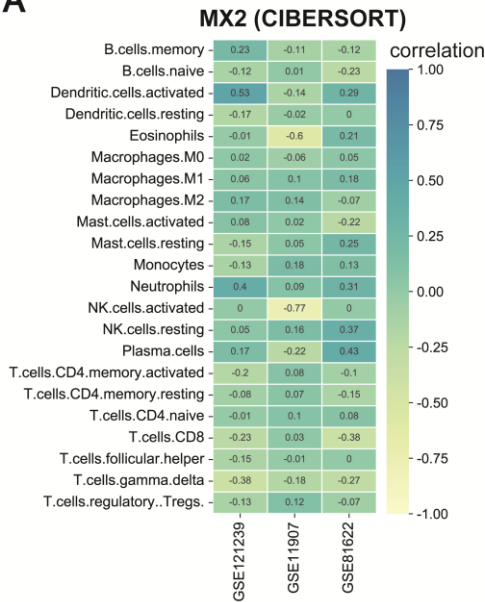

B

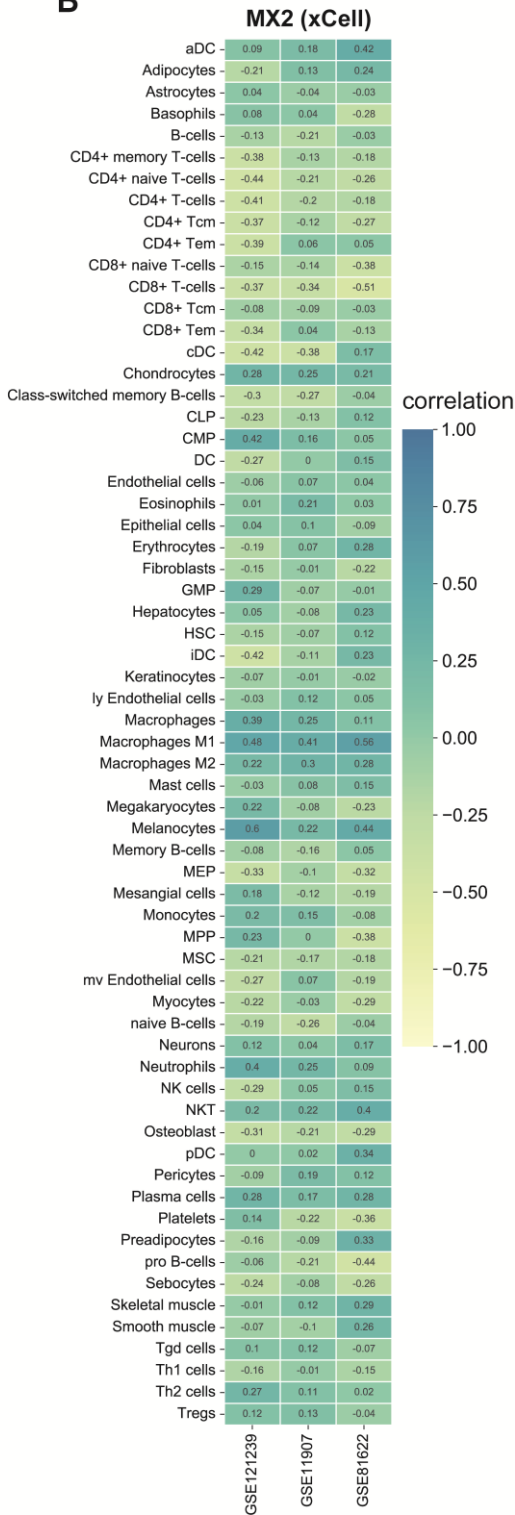

C

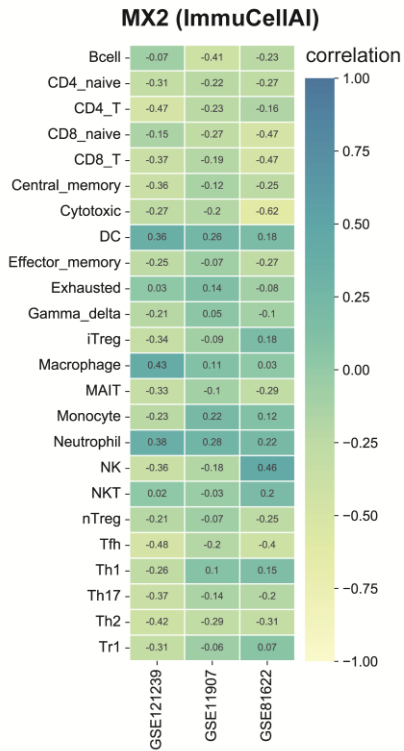

D

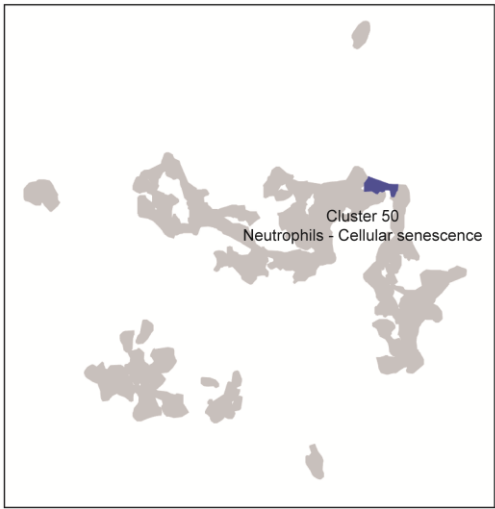

E

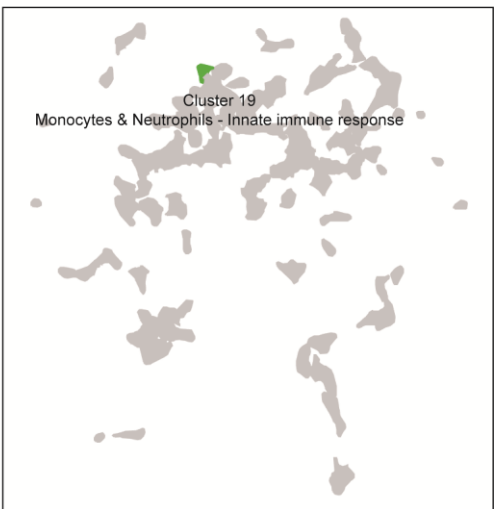

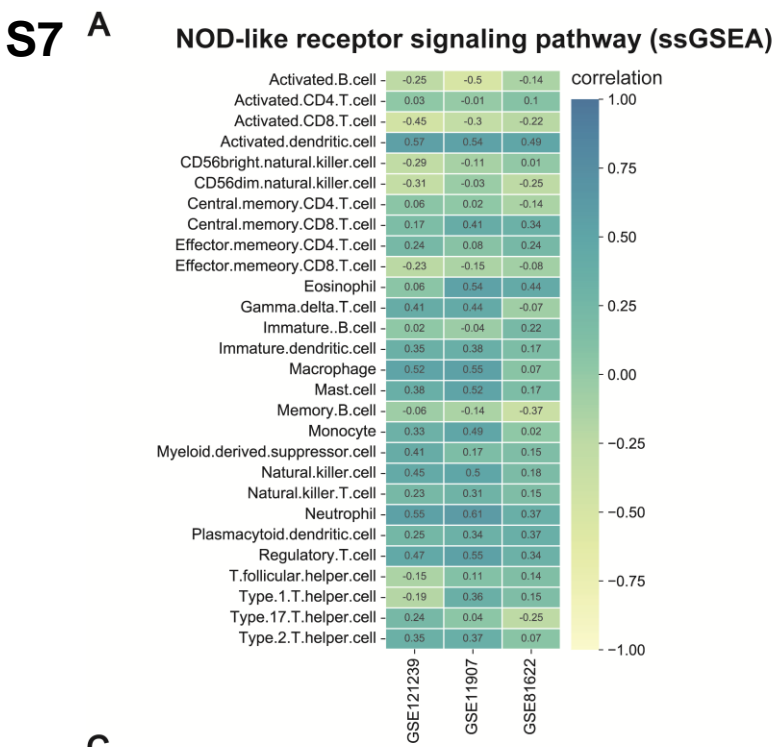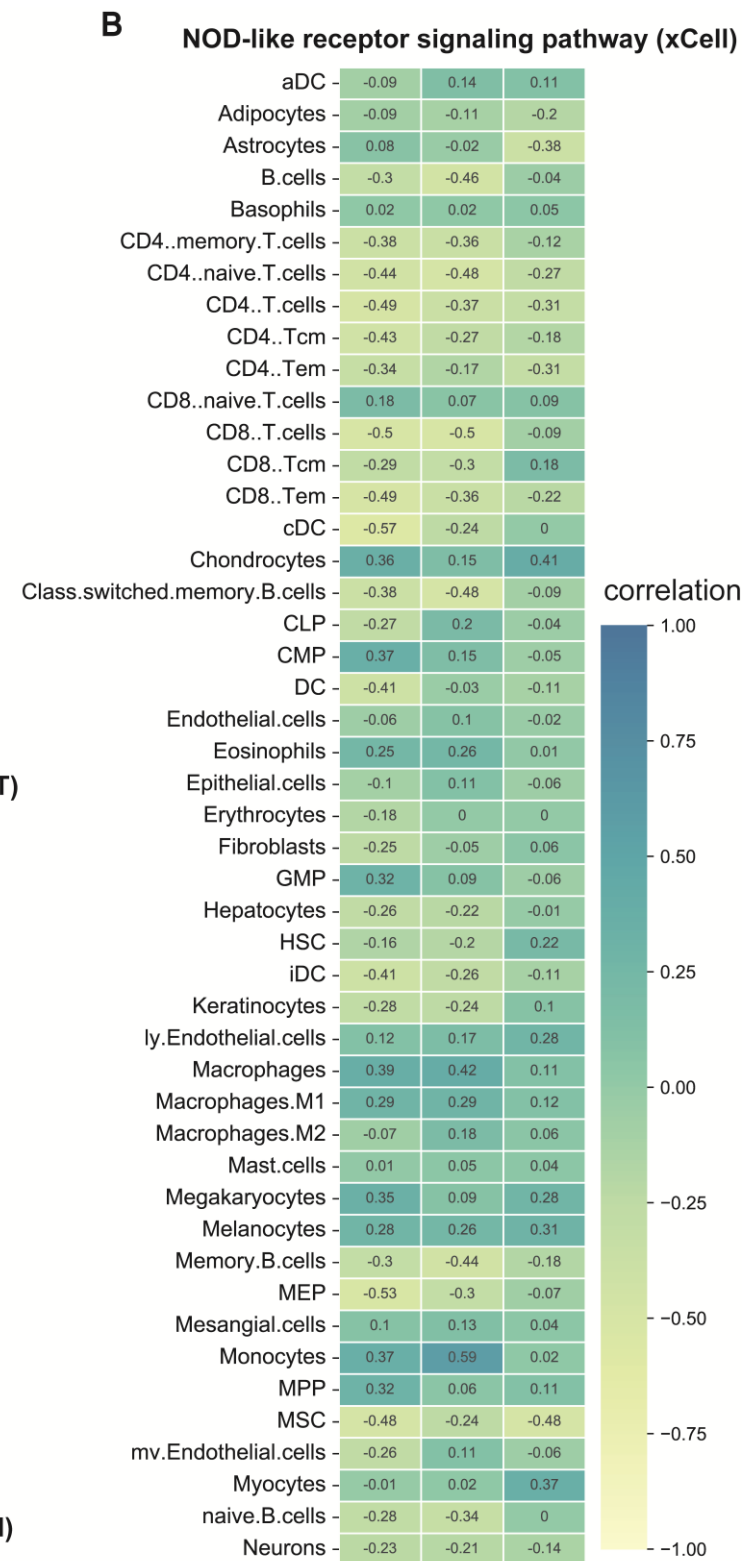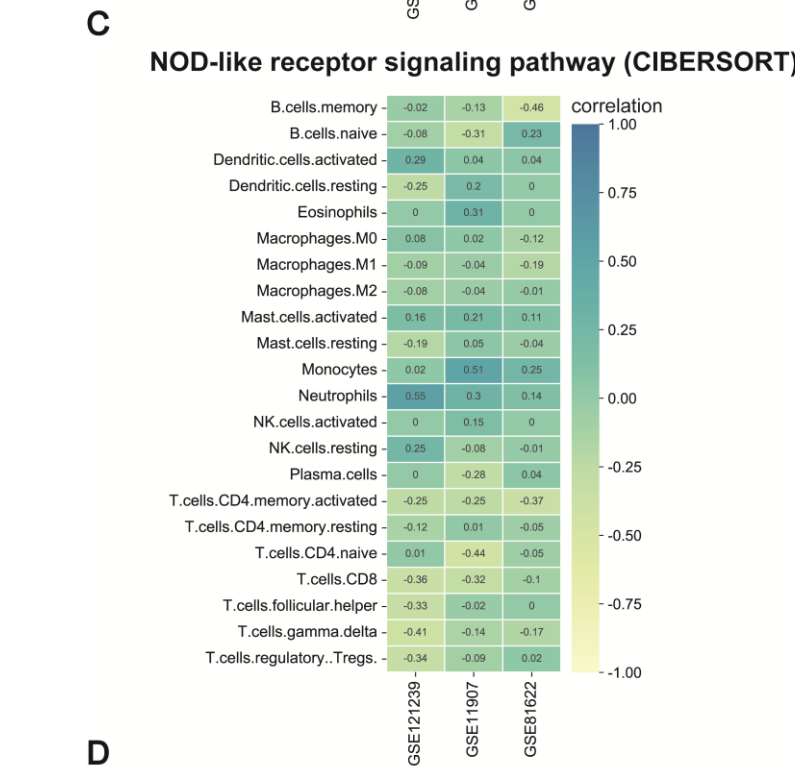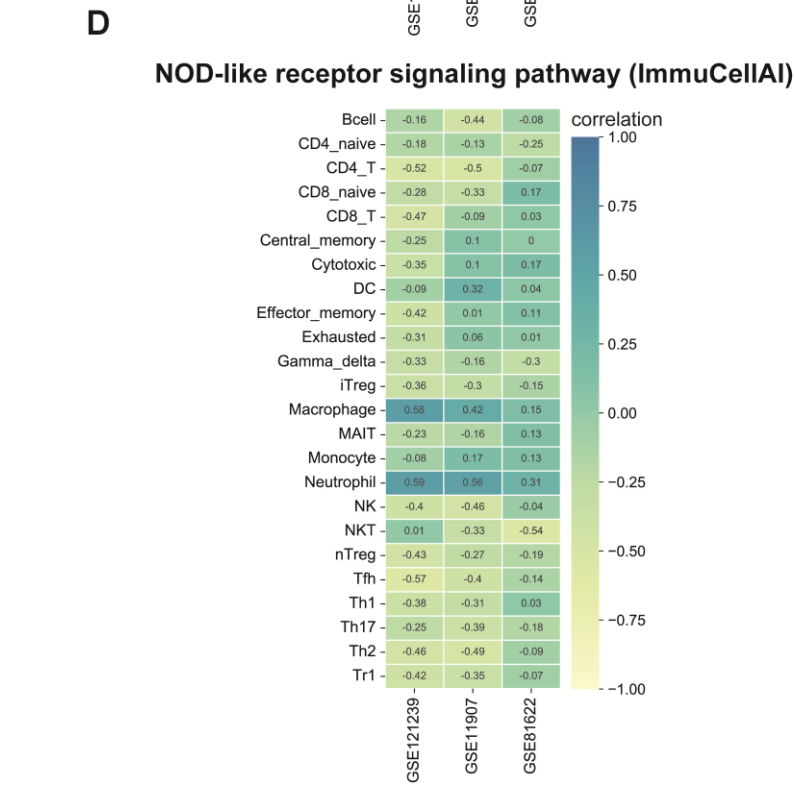

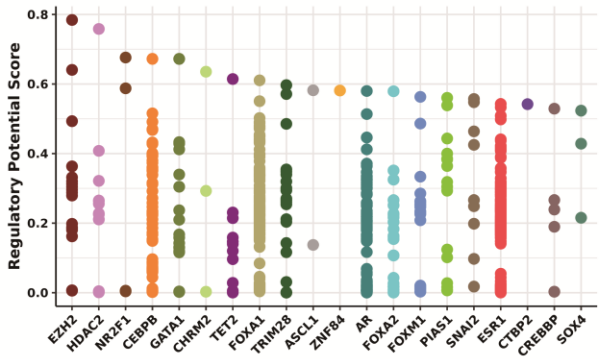

HERC5

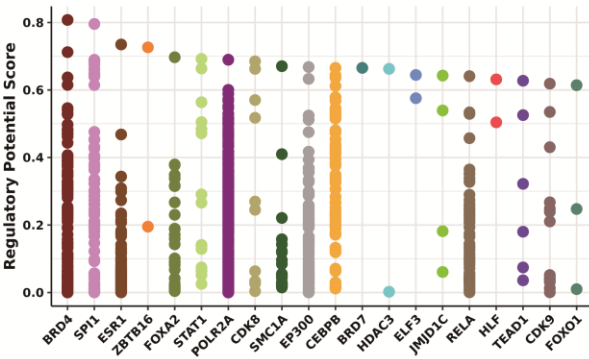

IFIT2

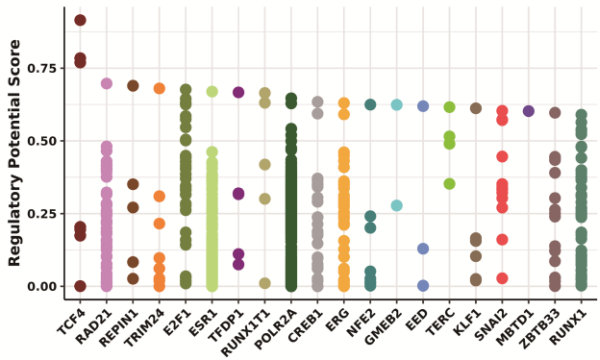

IRF7

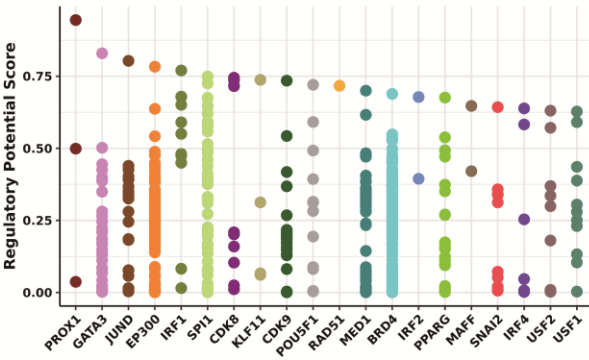

IFIT3

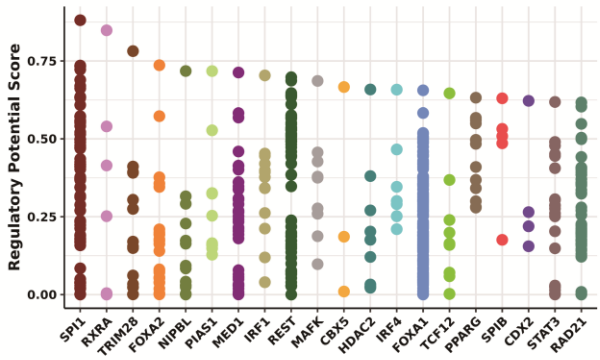

OAS1

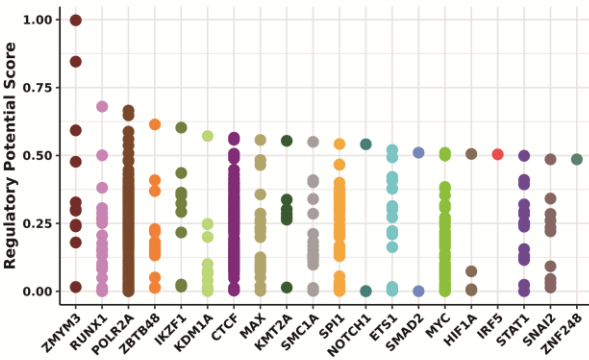

OAS2

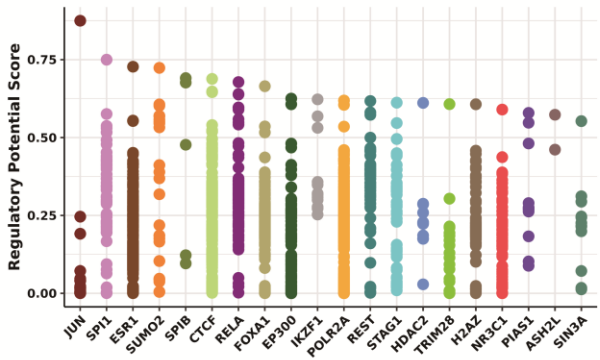

OAS3

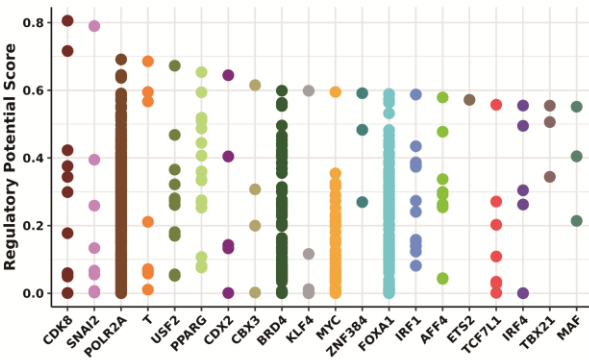

OASL

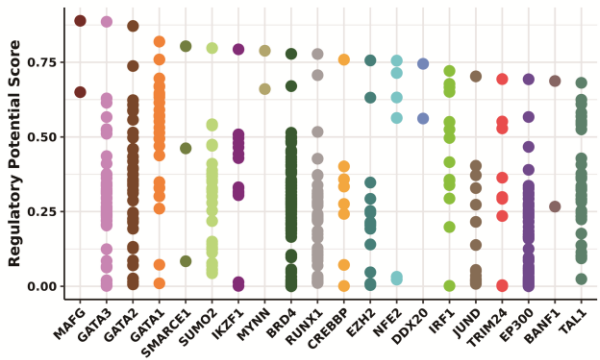

RSAD2

Supplement: Supplementary file 1 — The information of datasets and screening for MX2. [file DataSheet_1.zip › Supplementary materials (1)/Supplementary materials/Supplementary file 7.pdf]
